# Hypoxia-inducible factor-1α-deficient adipose-tissue macrophages produce the heat to mediate lipolysis of white adipose tissue through uncoupling protein-1

**DOI:** 10.1186/s42826-024-00224-4

**Published:** 2024-10-30

**Authors:** Gi-Sue Kang, Young-Eun Kim, Ho Rim Oh, Hye-Ju Jo, Seoyeon Bok, Yoon Kyung Jeon, Gi Jeong Cheon, Tae-Young Roh, Young-Tae Chang, Do Joong Park, G-One Ahn

**Affiliations:** 1 College of Veterinary Medicine, Seoul National University, Seoul, 08826 Korea; 2 Department of Life Sciences, Pohang University of Science and Technology (POSTECH), Pohang, 37673 Korea; 3 Department of Nuclear Medicine, College of Medicine, Seoul National University, Seoul, 03080 Korea; 4 Department of Pathology, College of Medicine, Seoul National University, Seoul, 03080 Korea; 5 Department of Molecular Medicine and Biopharmaceutical Sciences, Graduate School of Convergence Science and Technology, Seoul National University, Seoul, 08826 Korea; 6 College of Medicine, Cancer Research Institute, Seoul National University, Seoul, 03080 Korea; 7 Department of Life Sciences, Ewha Womans University, Seoul, 03760 Korea; 8 Department of Chemistry, POSTECH, Pohang, 37673 Korea; 9 Department of Surgery, College of Medicine, Seoul National University, Seoul, 03080 Korea

**Keywords:** Obesity, Uncoupling protein-1, Adipose-tissue macrophage, Heat, Lipolysis

## Abstract

**Background:**

Uncoupling protein 1 (UCP1) is a proton uncoupler located across the mitochondrial membrane generally involved in thermogenesis of brown adipose tissues. Although UCP1 is known to be strongly expressed in brown adipocytes, recent evidence suggest that white adipocytes can also express UCP1 under certain circumstances such as cold- or β-adrenergic receptor-stimulation, allowing them to acquire brown adipocyte-like features thereby becoming 'beige’ adipocytes.

**Results:**

In this study, we report that UCP1 can be expressed in adipose-tissue macrophages (ATM) lacking functional hypoxia-inducible factor-1 (HIF-1) and this does not require cold- nor β-adrenergic receptor activation. By using myeloid-specific *Hif-1α* knockout (KO) mice, we observed that these mice were protected from diet-induced obesity and exhibited an improved thermogenic tolerance upon cold challenge. ATM isolated from white adipose tissues (WAT) of these mice fed with high fat diet exhibited significantly higher M2-polarization, decreased glycolysis, increased mitochondrial functions and acetyl-CoA levels, along with increased expression of *Ucp1*, *peroxisome proliferator activated receptor-gamma co-activator-1a*, and others involved in histone acetylation. Consistent with the increased *Ucp1* gene expression, these ATM produced a significant amount of heat mediating lipolysis of co-cultured adipocytes liberating free fatty acid. Treating ATM with acetate, a substrate for acetyl-CoA synthesis was able to boost the heat production in wild-type or *Hif-1α*-deficient but not UCP1-deficient macrophages, indicating that UCP1 was necessary for the heat production in macrophages. Lastly, we observed a significant inverse correlation between the number of UCP1-expressing ATM in WAT and the body mass index of human individuals.

**Conclusions:**

UCP1-expressing ATM produce the heat to mediate lipolysis of adipocytes, indicating that this can be a novel strategy to treat and prevent diet-induced obesity.

**Supplementary Information:**

The online version contains supplementary material available at 10.1186/s42826-024-00224-4.

## Background

Obesity is a chronic inflammatory disease cause by imbalanced energy ingestion and expenditure frequently leading to other metabolic complications such as type 2 diabetes and cardiovascular diseases [1]. During obesity, adipocytes undergo a rapid expansion resulting in an insufficient blood flow, which in turn creates hypoxic regions within the white adipose tissue (WAT) [2, 3]. These changes in turn recruit immune cells such as macrophages (also known as adipose-tissue macrophages (ATM)), T cells, B cells, neutrophils, and mast cells into the adipose tissues further contributing to the chronic tissue inflammation and insulin resistance [4].

Although white adipocytes have traditionally been viewed as a passive repository for triglyceride accumulation [5], recent studies have suggested that these cells have a potential to turn into ‘brown-like’ adipocytes [6] when exposed to cold or catecholamine by expressing UCP1 (uncoupling protein-1) [7]. UCP1 is a proton uncoupler located in the inner mitochondrial membrane lowering the mitochondrial membrane potential thereby activating the respiratory chains and fatty acid oxidation dissipating the energy as heat in brown adipose tissues (BAT) [8]. UCP1 expression has been shown to be induced by cold-exposure or norepinephrine binding to β-adrenergic receptors, which increase the intracellular cyclic AMP and activate protein kinase A [7]. This then facilitates lipolysis liberating free fatty acid, overpassing the inhibitory effect of purine nucleotides on UCP thereby activating it in the inner mitochondrial membrane [7]. It has been reported that *Ucp1* transcription can be regulated by regulatory elements critical for both white and brown fat adipogenesis such as peroxisome proliferator-activated receptor (PPAR) and CCAAT/enhancer-binding protein (C/EBP) families and cAMP response-binding protein (CREB) [9]. Studies have also reported that hypoxia [10] or hypoxia-inducible factor-1 (HIF-1) [11] can negatively regulate UCP1 by suppressing PPAR.

Given the importance of tissue hypoxia created in WAT during obesity, a number of studies have reported the role of HIF in adipocytes in regulating obesity and insulin resistance. HIF is a heterodimeric transcription factor composed of an oxygen-sensitive HIF-α subunit (HIF-1α, -2α, and -3α) and a constitutively expressed HIF-1β subunit [12, 13]. In well-oxygenated condition, the oxygen-dependent degradation domain of HIF-α becomes hydroxylated by proline hydroxylases (PHD), which is then recognized and degraded by proteasomal degradation via von Hippel Lindau E3 ubiquitin ligase complex [14, 15]. In the absence of oxygen, PHD activities are inhibited, which makes HIF-α to be stabilized in the cytoplasm allowing its interaction with HIF-1β [15]. Given the early occurrence of tissue hypoxia in WAT during diet-induced obesity, a number of studies have demonstrated that HIF-1α in adipocytes can exacerbate insulin resistance and tissue inflammation [16]. Adipocyte specific deletion of *Hif-1α* in mice with Cre-recombinase under *aP2* promoter exhibited improved insulin sensitivity [17, 18], oxidative metabolism derived from expression of peroxisome proliferator activated receptor-gamma co-activator-1 [19], and tissue inflammation [20]. The mechanisms have been suggested that HIF-1α in adipocytes interferes with insulin signaling and glucose homeostasis by promoting nitric oxide and lactate production, which could interfere with the insulin signaling and glucose homeostasis [16]. In the study by Krishnan and colleagues [19], HIF-1α in adipocytes has been suggested to suppress β-oxidation of fatty acid through transcriptional repression of sirtuin 2, a member of the silent information regulator two class I family of NAD + -dependent deacetylases thereby inhibiting proliferator-activated receptor-γ coactivator-1α (PGC1α) expression and energy expenditure. However, there are also reports demonstrating contradictory findings such that the expression of dominant negative form of HIF-1α in adipocytes exhibited increased body weight gain and insulin resistance in mice [21].

WAT is highly heterogeneous complex tissue, composed of immune cells, endothelial cells, fibroblasts, neurons, and stem cells [5] and these cells may similarly experience the tissue hypoxia thereby activating HIF signaling. Adipose tissue macrophages (ATM) are known to be the most abundant immune cells in the obese adipose tissues [22]. In lean adipose tissues, ATM constitute less than 10% of the entire immune cell population, but in obese adipose tissues, this proportion can escalate up to 50% [22]. A number of reports have suggested that pro-inflammatory M1-polarized macrophages are the dominant population of ATM in the adipose tissues of obese individuals [23, 24]. In line with this, it has been reported that alternatively activated- and anti-inflammatory M2-polarized ATM protect mice from insulin resistance [25] and that PPAR-γ can regulate M2 marker expression, mitochondrial functions, and ATM recruitment to the adipose tissues [26]. Tissue-specific knockout (KO) mice targeting HIF pathways with myeloid-specific Lysozyme M promoter have demonstrated that HIF-1α regulates crown-like morphology [27], insulin sensitivity [27], infiltration [28], tissue inflammation [27, 28], and vascularity [27] in WAT. The role of HIF-2α seems somewhat unclear. Myeloid-specific *Hif-2α* KO mice fed with high fat diet have demonstrated no difference in the insulin sensitivity to their littermate control mice [29]. On the other hand, *Hif-2α*^+/−^ heterozygous mice injected with clodronate liposome to deplete macrophages have exhibited an improved glucose clearance [30]. In this study, we report our novel findings that UCP1 can be expressed in ATM of myeloid-specific *Hif-1α* KO mice generated with *hS100A8 (hMRP8)* promoter. We demonstrate that UCP1 expression in ATM of these mice does not require cold- nor catecholamine stimulation. Furthermore, UCP1-expressing ATM can generate a significant amount heat mediating lipolysis of WAT. We believe that UCP1 in ATM can be a novel therapeutic target to treat and prevent diet-induced obesity.

## Methods

### Mouse strains

All mice were maintained in specific pathogen-free environment and had access to water and food ad libitum. Floxed *Hif-1α* or *Hif-2α* mice were cross-bred with mice carrying the Cre recombinase gene under the h*MRP8* (human myeloid related protein 8) promoter as described previously [31, 32] and these mice were further crossed with *ApoE* null mice (stock no. 002052, Jackson laboratory, Bar Harbor, ME). Custom-designed atherogenic high-fat diet (HFD) containing 20% of fat (Cat. No. D101511) was purchased from Dyets Inc. (Bethlehem, PA) and given to 4 weeks (wk)-old mice ad libitum for up to 16–20 wk. To examine the Cre expression in tissues, *Cre *h*MRP8* mice were cross-bred with transgenic mice bearing loxP-STOP-loxP-eYFP under the ubiquitous *Rosa26* promoter (ROSA-eYFP; stock no. 006148, Jackson laboratory). All animal procedures were approved by the Institutional Animal Care and Use Committee (IACUC) at POSTECH (2019-0006) and Seoul National University (SNU-200224-2, 210524-5, and 211104–6).

### Cold challenge

Mice fed with high fat diet 12 wk were individually housed in a plastic cage (Tecniplast, Buguggiate, Italy) without bedding and placed in 4 °C cold room for 5 h. At each hour, mouse was held with one hand with scruffing position and measured for the core temperature by inserting a rectal temperature probe (Physitemp, Clifton, NJ) with its tip lightly coated with Vaseline™ (Unilever, United Kingdom).

### Staining of tissues/cells

Adipose tissues or the heart were harvested from animals, embedded in optical cutting temperature compound (OCT) (SAKURA, Finetek, Torrance, CA) and prepared as 8 μm frozen sections on microscopic slides. Slides were immersed in cold phosphate buffered saline (PBS) for 5 min, followed by post-fixation using 100% ice-cold acetone for 10 min. Sections were then incubated with rat/rabbit anti-mouse F4/80 antibodies (ab6640; Abcam, Cambridge, UK) or goat anti-mouse S100A8 (R&D systems, Minneapolis, MN) overnight at 4 °C followed by appropriate secondary antibodies including anti-rat Alexa 546 (Life technologies, Carlsbad, CA), anti-rabbit Alexa 488 (Life technologies), or anti-goat Alexa Fluor 546 (A11056; Life technologies) for 2 h at the room temperature. Slides were washed twice with PBS followed by mounting using ProLong Gold antifade mounting media with DAPI (Life technologies). For H&E staining, tissue sections were fixed with 10% formalin (Junsei chemical, Tokyo, Japan) incubated with hematoxylin (Sigma-Aldrich) for 3.5 min followed by eosin (Sigma-Aldrich) for 15 s. Stained slides of more than 5 random areas at 400 × magnification were analyzed with Zeiss Axio Scope (Carl Zeiss) with AxioVision program (v4.8). Oil Red O staining was performed with pre-adipocytes isolated from stromal vascular fraction. Isolated pre-adipocytes were differentiated by using 0.5 mM methylisobutylxanthine (Sigma- Aldrich), 1 μM dexamethasone (Sigma-Aldrich), 1 μM insulin (Sigma-Aldrich) in DMEM for 6 d. After 72 h incubation, fresh media containing 1 μM insulin was added every other day for 7 d. After differentiation, cells were fixed in 10% formalin for 30 min at 4 °C followed by incubation with 0.5% Oil Red O (Sigma-Aldrich) dissolved in 60% isopropanol (Sigma-Aldrich) for 10 min and washing with water. For quantification, stained cells were washed with 100% isopropanol, and the collected extract was subjected to the optical density measurements at 540 nm using Multiskan FC (Thermo Fisher Scientific). *En face* analysis of the aortic lesion was performed as described by others [33]. Briefly, 20 wk of high fat fed mice were euthanized with CO_2_ and cardiac perfused with warm PBS followed by 2% of low melt agarose perfusion. The whole aorta were dissected longitudinally under a dissecting microscope, carefully cut in half, pinned (Fine Science Tools, Foster City, USA) on P100 petri dish coated with dental wax. Tissues were then incubated with absolute propanediol for 1 min followed by Oil Red O incubation for 2 h, washed with distilled water 3 times, and taken for microphotographs for the quantification using image J software (National Institute of Health).

### Fluorescence-activated cell sorting (FACS) analyses

Stromal vascular fraction was prepared as described by others [34]. Cells were incubated in FACS buffer (PBS with 3% fetal bovine serum (FBS) supplement) containing APC anti-F4/80 (eBioscience, San Diego, CA), PE-Cy7 anti-CD11b (eBioscience), Pacific Blue-CD11b (eBioscience), PE anti-CD11c (BioLegend, San Diego, CA), or Alexa Fluor 488 anti-CD206 (BioLegend) antibodies. After 30 min on ice, cells were washed in FACS buffer, introduced to MoFlo-XDP (Beckman Coulter, Pasadena, CA) to sort/analyze with FlowJo software 10.4.1 (Tree Star, Inc).

### Quantitative real time-polymerase chain reaction (qRT-PCR)

Total RNA was extracted using TRIZOL according to the manufacturer’s protocol (Invitrogen, Carlsbad, CA). cDNA was synthesized as described previously [35] and introduced to Step One Plus (Applied Biosystems, Foster city, CA). Primer sequences are listed in Supplementary Table 1.

### Mitochondrial function measurements

ATP levels in ATM were measured according to the manufacturer’s protocol (Abcam). Briefly, cells were lysed with ATP assay buffer, and insoluble fractions were separated and discarded by centrifugation. Supernatants were deproteinized with perchloric acid (Junsei chemical) and neutralized with potassium hydroxide (Daejung). After centrifugation at 13,000 g for 15 min at 4 °C, supernatants were collected, added with ATP reaction mix, and measured by SPECTROstarNano (BMG LABTECH, Ortenberg, Germeny). Oxygen consumption rates were measured using Oxytherm system with Clark oxygen electrode (Hansatech, King’s Lynn, U.K). ATM were placed in a chamber with a magnetic bar and were measured oxygen consumption for 5 min at the room temperature. Data were acquired when oxygen concentrations starting to decline according to the manufacturer’s protocol.

### Western blot

Tissues or cells were lysed in radioimmunoprecipitation assay buffer (RIPA) containing protease inhibitors (Calbiochem). Proteins were quantified the bicinchoninic acid (BCA; Thermo Fisher Scientific) assay and loaded on NuPAGE 12% Bis–Tris gel (Life technology), transferred to PVDF membrane (0.2 μM; Biorad, Hercules, CA), and incubated with primary antibodies such as rabbit anti-mouse HIF-1α (NB100-449; Novus Biologicals, Littleton, CO), p38 (PA5-27,831; Thermo Fisher Scientific), phospho-p38 pThr180 + Tyr182 (MA5-15,182; Thermo Fisher Scientific), HSL (4107S; Cell Signaling Technology; CST, Danvers, MD), phospho-HSL Ser660 (4126S; CST), or Actin (69,100; MP biomedicals) overnight at 4 °C followed by washing with TBST (tris-buffered saline with Tween20). The membrane was further incubated with appropriate secondary antibodies conjugated with horse radish peroxidase (Ab6721; Abcam) for 2 h at the room temperature followed by development with enhanced chemiluminescence substrate (32,106; Thermo Fisher Scientific), or supersignal west dura extended duration substrate (34,076, Thermo Fisher Scienticfic) using CL-X Posure film (34,091; Thermo Fischer Scientific).

### ATM staining with mitotracker or ERthermAC

Isolated ATM were placed in a chamber slide (Corning) and incubated for 12 h in DMEM supplemented with 10% FBS at 37 °C to allow attachment onto the slide. Cells were then fixed105 with 4% paraformaldehyde (Daejung) for 15 min followed by PBS washing and staining with 100 nM MitoTrackerTM Red CMXRos (Invitrogen, Thermo Fisher Scientific) for 30 min at the room temperature. For ERthermAC staining, the sorted ATM or BMDM were placed in chamber slides and stained with 250 nM ERthermAC for 30 min at 37 °C when cells were attached. Slides were washed twice with warm PBS followed by fixation with 4% PFA for 30 min. Slides were mounted with ProLong Gold antifade mounting media with DAPI (Life technologies). For measurement of ERthermAC signal intensity, cells were allowed to attach in 96 well plates and stained with 250 nM ERthermAC for 30 min at 37 °C followed by washing with PBS. Cells were then incubated with DMEM media and output was measured on a microplate reader (Ex/Em = 510 / 590 nm; TECAN, Männedorf, Switzerland).

### Enzyme-linked immunosorbent assay (ELISA)

To measure the insulin, plasma was collected from mice fed with high-fat diet for 12 wk using insulin ELISA kit (90,080; Crystal Chem INC. Elk Grove Village, IL).

### Free fatty acid (FFA) and triacyl glyceride (TG) measurement

For FFA or TG measurements, plasma was obtained from mice as above. WAT or cells were harvested and prepared as lysate. With these samples, FFA or TG were measured using FFA Assay Kit (ab65341; Abcam) or TG Assay Kit (ab65336; Abcam) according to the manufacturers’ instructions.

### Metabolic parameter measurements

For glucose tolerance tests, mice fed with high fat diet were fasted for 8 h followed by an intraperitoneal injection of glucose (1 g/kg). Blood glucose levels were measured using Glucolab glucometer (Infopia, Anyang, Korea). To measure daily food and water intake or feces and urine production, individual mouse was housed in a metabolic cage for 5 consecutive days. At each day, the amount of food and water consumed was measured, and feces and urine were collected in 50 ml conical tubes and weighed. Data from the 2nd day measurement were analyzed. To measure the respiratory exchange ratio, an individual mouse was placed in a plastic chamber equipped with a treadmill with an electric shock bar (Comprehensive Lab Animal Monitoring System chambers). Mice were forced to run until exhausted by increasing the speed and slope of the treadmill every 3 min. CO_2_ production and O_2_ consumption were recorded and analyzed using Oxymax program (Columbus Instruments; Columbus, Ohio).

### WAT analysis from human subjects

Human subject study was approved by Seoul National University Hospital Institutional Review Board (IRB No. H-2002-116-1103). Written informed consent was obtained for all patients before the surgery. Tissues were removed and immediately fixed in 4% formalin (BPP-9004, Tech & Innovation, chuncheon-si, Korea) overnight, and made as paraffin embedded blocks. Serial tissue sections were made at 4 μm thickness, deparaffinized with xylene, rehydrated by serial ethanol dipping followed by peroxidase blocking with 0.5% H_2_O_2_ for 30 min. Antigen retrieval and permeabilization were performed using 10 mM sodium citrate (pH 6.0) with heat and 0.5% Triton X-100 in Tris buffered saline (TBS), respectively. Slides were then stained using rabbit isotype antibody (02-6102, Invitrogen), mouse isotype antibody (31,903, Invitrogen), UCP1 antibody 1:500 (PA1-24,894, Invitrogen), or CD68 antibody 1:50 (sc-20060, Santa Cruz biotechnology, Dallas, TX). Biotinylated horse radish peroxide secondary antibodies (anti-rabbit IgG, BA-1100, Vector laboratories, Burlingame, CA; anti-mouse IgG, BA-9200, Vector laboratories) were applied to the matched with primary antibody species at 1:250 dilution for 1 h followed by exposure of 3, 3’-diaminobenzidine (DAB) substrate (SK-4100, Vector laboratories). For counterstaining, hematoxylin (S3309, Dako, Santa Clara, CA) was treated for 20 s. Finally, stained slides were dehydrated and mounted in Permount mounting medium (SP15-100, Thermo Fisher Scientific).

### Quantification and statistical analysis

Data were analyzed by two-tailed Student’s *t*-test, one- or two-way ANOVA using GraphPad Prism software version 10 (GraphPad Inc, La Jolla, CA). *P values* less than 0.05 were considered to be statistically significant.

## Results

### Myeloid-specific *Hif-1α* knockout (KO) mice were protected from diet-induced obesity but not from atherosclerosis

**Fig. 1:**
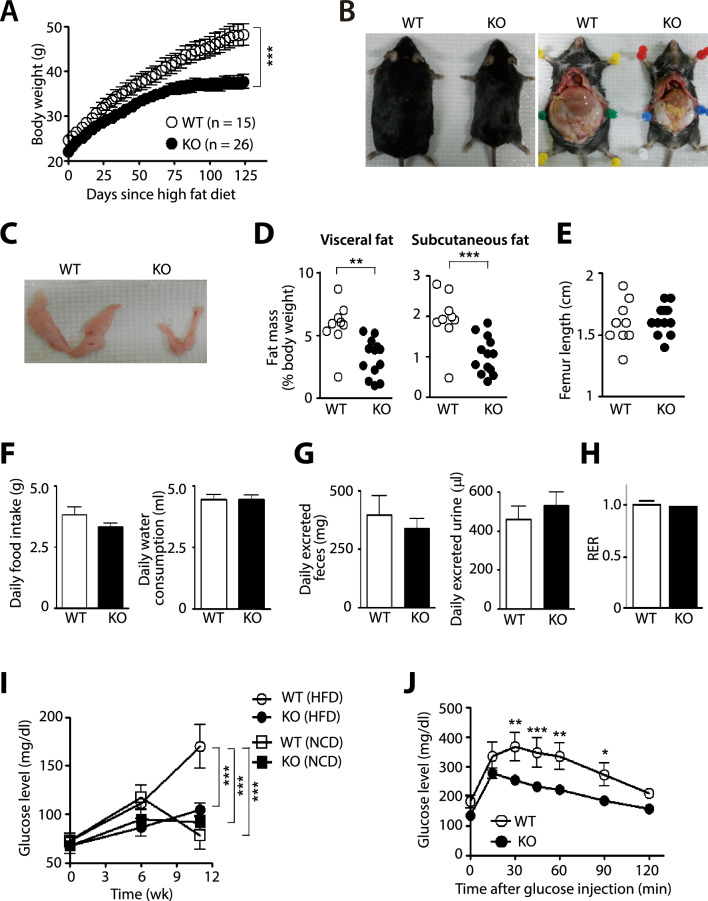
Myeloid-specific *Hif-1α* knockout mice exhibit an anti-obese phenotype. **A** Body weight changes in WT or myeloid-specific *Hif-1α* knockout (KO; h*MRP8cre*^+^; *Hif-1α*^fl/fl^; *ApoE*^−/−^) mice upon HFD. *** indicates *P* < 0.001 by two-way ANOVA. **B** Photographs of WT and myeloid-specific *Hif-1α* KO mice after 20 wk of HFD. **C** Photographs of isolated eWAT. **D** Quantification of the visceral (left) and subcutaneous (right) fat mass obtained from mice in (**B**) normalized to their body weight. ** and *** indicate *P* < 0.01 and 0.001, respectively, by Student’s *t*-test. **E** Femur lengths of mice in (**B**). **F, G** Daily food (left) and water (right) consumption (**F**) or daily feces (left) and urine (right) excretion (**G**) in setting of (**B**). Data are the mean ± s.e.m. from at least 5 animals per group. **H** Respiratory exchange ratio (RER) determined as VCO_2_/VO_2_ from treadmill exercise of WT or myeloid-specific *Hif-1α* KO mice after 20 wk of HFD. **I** Fasting blood glucose level in mice fed with NCD (n = 5 for WT mice; n = 6 for myeloid-specific *Hif-1α* KO mice) or HFD (n = 6 for WT mice; n = 6 for myeloid-specific *Hif-1α* KO^−^ mice). *** indicates *P* < 0.001 by two-way ANOVA. **J** Glucose tolerance test in WT (n = 4) or myeloid-specific *Hif-1α* KO mice (n = 10) after 12wk of HFD. *, **, and *** indicate *P* < 0.05, 0.01, and 0.001, respectively, by two-way ANOVA. Symbols and error bars are the mean ± S.E.M

We generated myeloid-specific *Hif-1α* KO mice by crossing mice expressing Cre-recombinase under hMRP8 myeloid-specific promoter with mice having *Hif-1α* floxed mice (h*MRP8*Cre^+^; *Hif-1α*^fl/fl^) and further crossed them to *ApoE* null background (h*MRP8*Cre^+^; *Hif-1α*^fl/fl^; *ApoE*^−/−^; hereafter denoted as myeloid-specific *Hif-1α* KO mice) to initially examine a role of HIF-1α in myeloid cells in atherosclerosis. We challenged these mice to atherogenic diet containing 20% fat for 20 wk [36]. We observed that there was no difference in atherosclerotic plaque formation between myeloid-specific *Hif-1α* KO mice and their littermate control (h*MRP8*Cre^−^; *Hif-1α*^fl/fl^; *ApoE*^−/−^; hereafter denoted as wild type (WT) mice), as determined by Oil Red O staining in the aorta (Supplementary Fig. 1A) or aortic sinus (Supplementary Fig. 1B). Immunostaining for myeloid cells by using S100A8 antibodies in the aortic sinus further showed no difference in the myeloid cell infiltration between the two groups (Supplementary Fig. 1C). Interestingly however, we observed that myeloid-specific *Hif-1α* KO mice gained significantly less body weight compared to WT upon high fat diet (HFD; Fig. 1A). To examine whether this phenotype was due to *ApoE* deficiency, we fed the myeloid-specific *Hif-1α* KO or WT mice in *ApoE*^+/+^ background and observed similar findings (Supplementary Fig. 1D), suggesting that the anti-obese phenotype in the myeloid-specific *Hif-1α* KO mice was not due to *ApoE* deficiency. Furthermore, we found that this effect was HIF-1α-specific because myeloid-specific *Hif-2α* KO (*hMRP8*Cre^+^; *Hif-2α*^fl/fl^) mice exhibited a similar body weight gain to their WT counterparts upon HFD (Supplementary Fig. 1E). Next, we examined whether hMRP8 was specifically expressed in adipose tissue macrophages (ATM) by performing immunostaining of WAT from the Cre-bearing offspring mouse obtained from *hMRP8Cre*^+^ mice cross bred with reporter mice harboring targeted mutation of the Gt(ROSA)26Sor locus with a loxP-flanked STOP cassette preventing CAG promoter-driven enhanced yellow fluorescent protein (eYFP) transcription. We observed eYFP^+^ staining only in WAT of mice bearing *Cre* gene (Supplementary Fig. 1F) but not those of *Cre*-negative mice (Supplementary Fig. 1F).

At 20 wk of high fat feeding, we dissected these mice and observed significantly reduced WAT mass in myeloid-specific *Hif-1α* KO mice compared to those in WT mice (Fig. 1B–D). To exclude a possibility whether the reduced fat mass in myeloid-specific *Hif-1α* KO mice may be associated with possible developmental defects, we measured the femur length in these mice and observed that there was no difference in the femur length between myeloid-specific *Hif-1α* KO and WT mice (Fig. 1E). We further confirmed that there was no difference in adipogenicity of pre-adipocytes between myeloid-specific *Hif-1α* KO mice and their WT compartments (Supplementary Fig. 1G). We next measured the amount of daily food and water consumption (Fig. 1F), feces and urine production (Fig. 1G), respiratory exchange ratio (RER; Fig. 1H) and observed no differences in these parameters between myeloid-specific *Hif-1α* KO and WT mice (Fig. 1F–H). Interestingly, fasting glucose levels in myeloid-specific *Hif-1α* KO mice fed with HFD were significantly lower those in WT mice (Fig. 1I) and these levels were comparable to myeloid-specific *Hif-1α* KO or WT mice fed with normal chow diet (NCD; Fig. 1I). Consistent with these results, glucose tolerance was significantly improved in myeloid-specific *Hif-1α* KO mice fed with HFD than those in WT counterpart (Fig. 1J).

### Enhanced lipolysis of WAT and improved thermogenic tolerability in myeloid-specific *Hif-1α* KO mice fed with HFD

**Fig. 2:**
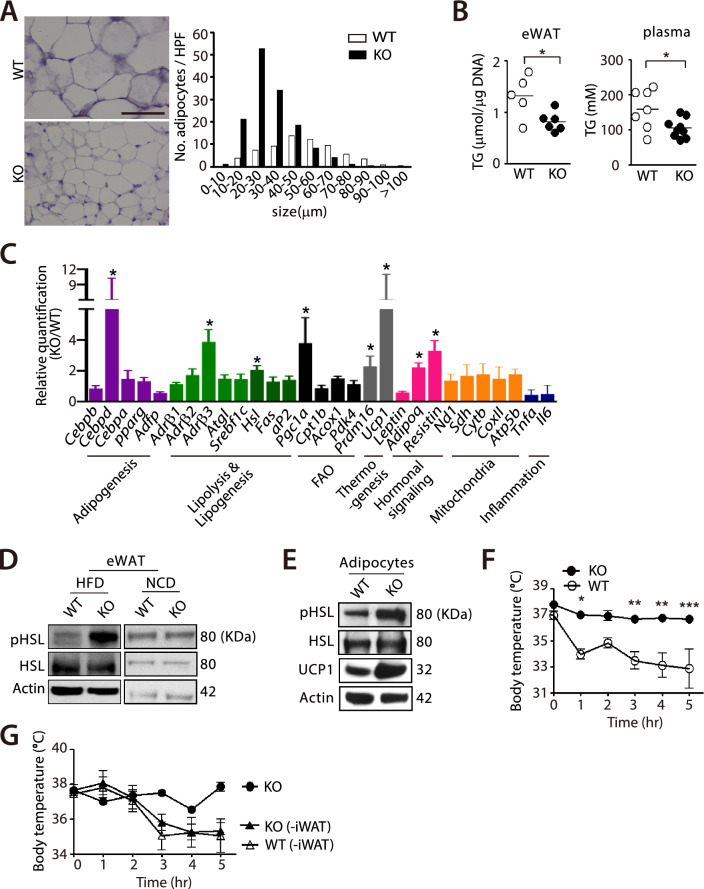
Increased lipolysis and thermotolerance in myeloid-specific *Hif-1α* KO mice fed with HFD. **A** Representative hematoxylin and eosin stained sections (left) and the number of adipocytes counted per high power field (HPF; right) of the eWAT from WT or myeloid-specific *Hif-1α* KO mice fed with HFD for 12 wk. **B** Triglyceride (TG) levels in eWAT (left) or in plasma (right) of WT or myeloid-specific *Hif-1α* KO mice in (**A**). Mice were fasted for 8 h prior to the sample collection. * indicates *P* < 0.05 by Student’s t-test. **C** Gene expression changes in eWAT of mice in setting of (**A**). * indicates an increased gene expression change by more than twofold. **D, E** Western blot analyses for phosphorylated HSL (pHSL), HSL and/or UCP1 in WAT (**D**) or adipocytes (**E**) derived from WAT of WT or myeloid-specific *Hif-1α* KO mice after 12 wk of NCD or HFD. Actin was used as a loading control. **F** Core body temperature of WT (n = 6) or myeloid-specific *Hif-1α* KO mice (n = 5) fed with HFD upon cold challenge for 5 h. *, ** and *** indicate *P* < 0.05, 0.01 and 0.001, respectively, by two-way ANOVA. **G** The core body temperature of myeloid-specific *Hif-1α* KO mice (n = 2), WT mice surgically removed iWAT (n = 4) or myeloid-specific *Hif-1α* KO mice surgically removed for iWAT (n = 5) that had been fed with HFD for 12 wk upon cold change as in (**F**). Mice were allowed to recover 2 wk following the surgery before fed with HFD. Symbols and error bars are the mean ± S.E.M

To investigate how myeloid-specific *Hif-1α* KO mice were protected from the diet-induced obesity, we examined histology of epididymal white adipose tissue (eWAT). We observed significantly reduced size of adipocytes in eWAT from myeloid-specific *Hif-1α* KO mice compared to those in WT mice (Fig. 2A). We next measured triglyceride (TG) levels to examine dyslipidemia and found that myeloid-specific *Hif-1α* KO mice exhibited significantly decreased TG levels in both eWAT and plasma than WT mice (Fig. 2B). These results may indicate that myeloid-specific *Hif-1α* KO mice may have an increased level of adipose tissue breakdown, a process known as lipolysis. To prove this, we isolated eWAT and examined expression of various genes involved in lipolysis. We observed that *β3-adrenergic receptor* (*Adrβ3*), *peroxisome proliferator activated receptor gamma co-activator-1α* (*Pgc1α*), and *Ucp1* were significantly increased in eWAT (Fig. 2C) and inguinal WAT (iWAT; Supplementary Fig. 2A) of myeloid-specific *Hif-1α* KO mice fed with HFD. Consistent with the gene expression changes, we also observed an increased expression of activated (phosphorylated) hormone sensitive lipase (HSL), a rate limiting enzyme mainly responsible for breaking down TG [37] in eWAT (Fig. 2D) or adipocytes isolated from eWAT (Fig. 2E) of myeloid-specific *Hif-1α* KO mice fed with HFD. Similar increase in the phosphorylated HSL (pHSL) was observed in iWAT of myeloid-specific *Hif-1α* KO mice (Supplementary Fig. 2B).

Based on the increased expression in thermogenic genes in WAT of myeloid-specific *Hif-1α* KO mice, we performed cold challenge to these mice and measured their core body temperature at every hour up to 5 h. We found that myeloid-specific *Hif-1α* KO mice fed with HFD were significantly more resistant (Fig. 2F) whereas WT mice exhibited a steady drop in the core body temperature (Fig. 2F) upon cold challenge. To determine whether WAT was responsible for the thermogenic tolerability in myeloid-specific *Hif-1α* KO mice, we surgically removed iWAT, which has been known to undergo browning process upon cold-challenge [38], and challenged them to the cold. We found that myeloid-specific *Hif-1α* KO mice removed for iWAT now completely lost the thermogenic tolerability (Fig. 2G) such that their body temperature changes were comparable to those of WT mice removed for iWAT (Fig. 2G).

### HIF-1α-deficient ATM exhibit increased M2 polarization and mitochondrial functions

**Fig. 3:**
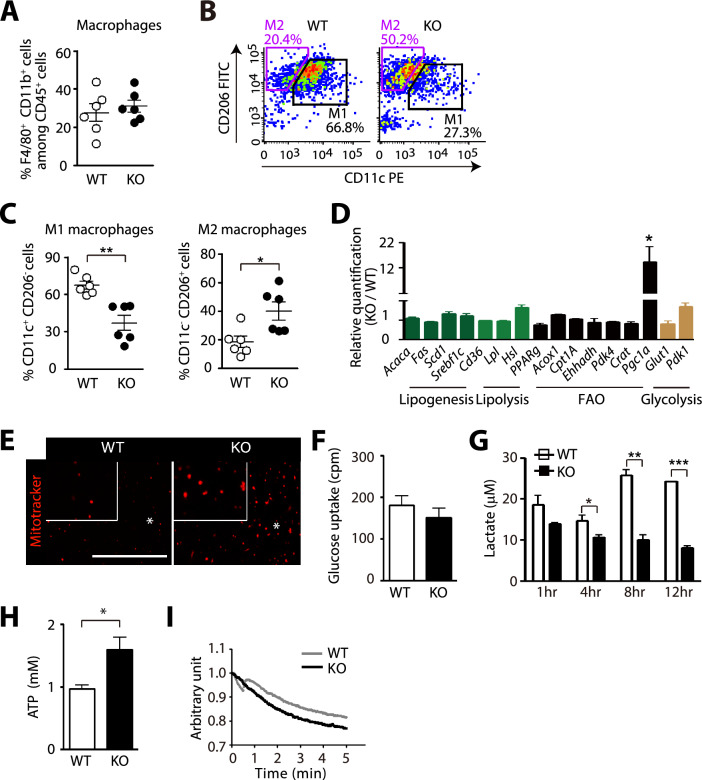
ATM deficient for HIF-1α exhibit increased M2 polarization and mitochondrial functions. **A** Percentages of F4/80 + CD11b + cell adipose tissue macrophages (ATM) in eWAT. **B, C** Gating strategy (**B**) and portion of F4/80^+^CD11b^+^CD11c^+^ M1 macrophages (**C**, left), F4/80 + CD11b + CD206 + M2 macrophages (**C**, right) ATM in eWAT of WT or myeloid-specific *Hif-1α* KO mice fed with HFD for 12 wk, analyzed by FACS. **D** Gene expression changes in FACS-sorted ATM from WT or myeloid-specific *Hif-1α* KO mice fed with HFD for 12 wk. * indicates an increased gene expression change by more than twofold. **E** Representative images of the sorted ATM as in (**C**) stained with Mitotracker Red. Scale bar denotes 100 μm. **F** Glucose uptake into the sorted ATM prepared as in (**C**). **G** Lactate measurement in the supernatant from the sorted ATM prepared as in (**C**). Supernatant was harvested at 1, 4, 8, and 12 h of incubation. **H, I** ATP levels (**H**) and oxygen consumption rate (**I**) in the sorted ATM as in (**C**). *, ** and *** indicate *P* < 0.05, 0.01 and 0.001, respectively, by Student’s *t*-test

Since we disrupted HIF-1α only in cells of the myeloid lineage, we examined immunophenotype of ATM in these mice. We found that total number of ATM in eWAT was similar between myeloid-specific *Hif-1α* KO mice and WT mice fed with HFD (Supplementary Fig. 2C, Fig. 3A). However, myeloid-specific *Hif-1α* KO mice had significantly increased M2-polarized ATM (CD11b^+^ F4/80^+^ CD11c^−^ CD206^+^; Fig. 3B, C) and decreased M1-polarized macrophages (CD11b^+^ F4/80^+^ CD11c^+^ CD206^−^; Fig. 3B, C) compared to WT mice. By sorting ATM from eWAT of these mice, we found that ATM from myeloid-specific *Hif-1α* KO mice had significantly increased *Pgc1α* gene expression among others (Fig. 3D). Consistent with the fact that PGC1α is known to play an essential role in mitochondrial biogenesis [39], we observed an increased staining of mitotracker in ATM isolated from myeloid-specific *Hif-1α* KO mice (Fig. 3E). Because HIF-1 regulates many key enzymes involved in glycolysis [40], we hypothesized that ATM deficient for HIF-1α may be impaired in glycolysis. Although glucose uptake was comparable between the two groups (Fig. 3F), ATM deficient for HIF-1α exhibited a significantly decreased lactate production (Fig. 3G) whereas ATP production (Fig. 3H) and oxygen consumption (Fig. 3I) were significantly increased. These results thus suggest that HIF-1α-deficient ATM had impaired glycolysis while mitochondrial functions were enhanced.

### HIF-1α-deficient ATM generate the heat through UCP1 to mediate lipolysis of adipocytes

**Fig. 4:**
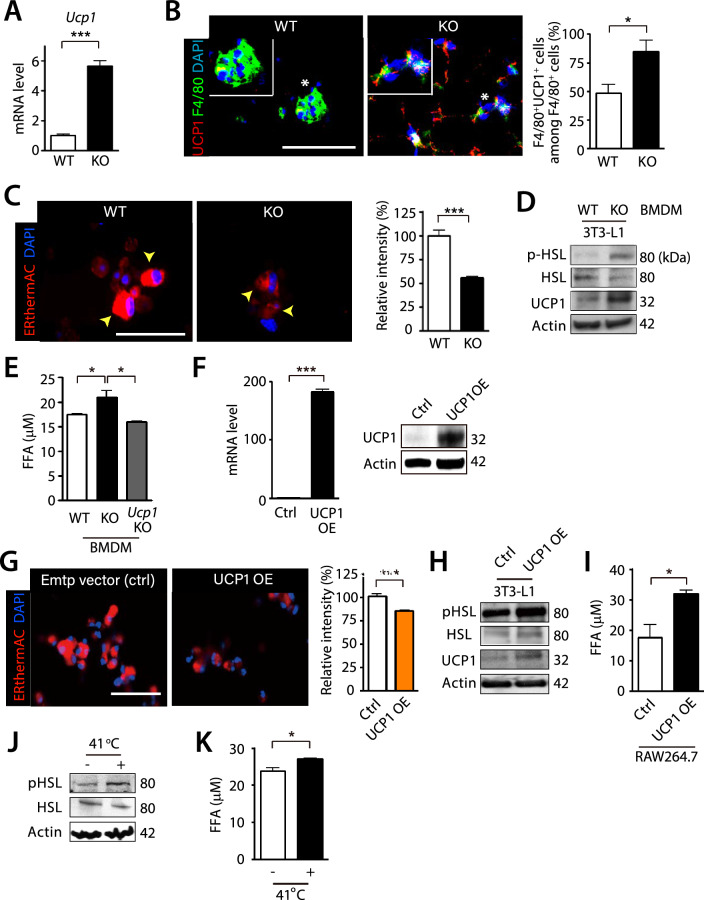
UCP1 is necessary and sufficient for the heat production in ATM mediating lipolysis. **A** mRNA expression of *Ucp1* in sorted ATM in setting of Fig. 3**C**. **B** Immunofluorescence staining (left) of F4/80 (green) and UCP1 (red) in eWAT obtained from WT or myeloid-specific *Hif-1α* KO mice fed with HFD for 12 wk. Nuclei are shown in blue with DAPI counterstaining. Inserts are magnified regions where asterisks are marked. Scale bar denotes 100 μm. Quantification of F4/80^+^UCP1^+^ cells among the total F4/80^+^ cells is shown on the right. Data are the mean ± s.e.m. from n ≥ 5 mice per group. **C** ERthermAC (red) staining in the sorted ATM in mice as in (**A**). Yellow arrowheads indicate cells with ERthermAC staining. Quantitative analysis of ERthermAC intensity is shown in the right bar graph. **D** Western blot analysis for pHSL, HSL, and UCP1 of 3T3-L1 adipocytes (5 × 10^5^ cells) directly co-cultured with WT or myeloid-specific *Hif-1α* KO mice BMDM (0.5 × 10^5^ cells) for 24 h. **E** FFA levels in the supernatant of 3T3-L1 adipocytes (5 × 10^5^ cells) directly co-cultured with WT, myeloid-specific *Hif-1α* KO mice, or *Ucp1* KO mice BMDM (0.5 × 10^5^ cells) for 24 h. **F** mRNA (left) and protein expression (right) of UCP1 in RAW264.7 cells after transfection of UCP1 overexpression vector. **G** ERthermAC (red) staining in RAW264.7 cells of (**F**). Nuclei of (**C**) and (**G**) are shown in blue with DAPI counterstaining. Scale bars of (**C**) and (**G**) denotes 50 μm. **H**,** I** Western blot analysis of pHSL, HSL, and UCP1 (**H**) or cellular FFA secretion (**I**) in 3T3-L1 adipocyte co-cultured with macrophages from (**F**). **J**-**K** Western blot of pHSL and HSL (**J**) or cellular FFA levels (**K**) in 3T3-L1 adipocytes exposed to 37 °C or 41 °C

We next determined how ATM deficient for HIF-1α could mediate lipolysis of WAT. Because PGC1α can activate UCP1 by binding to peroxisome proliferator responsive elements of *UCP1* promoter [41], we examined whether *Ucp1* might be expressed in ATM of myeloid-specific *Hif-1α* KO mice. By sorting ATM from eWAT of myeloid-specific *Hif-1α* KO mice fed with HFD, we observed indeed that *Ucp1* expression was significantly increased in myeloid-specific *Hif-1α* KO mice than WT mice (Fig. 4A). Furthermore, immunostaining of eWAT also revealed that there were increased numbers of UCP1^+^ ATM in myeloid-specific *Hif-1α* KO mice than WT mice (Fig. 4B). Given the role of UCP1 in the heat production [7], we hypothesized that ATM from myeloid-specific *Hif-1α* KO mice with an increased *Ucp1* expression would also be able to generate heat. To test this hypothesis, we utilized ERthermAC, a thermo-sensitive fluorescent dye that has been reported to successfully measure the heat generation in brown adipocytes by accumulating in the endoplasmic reticulum, with which their membranes are fused with mitochondrial membrane [42]. We observed that ATM from myeloid-specific *Hif-1α* KO mice exhibited a significantly dimmer ERthermAC fluorescence intensity than those from WT mice (Fig. 4C), indicating that ATM from myeloid-specific *Hif-1α* KO mice could indeed generate the heat.

To examine whether UCP1 in macrophages was responsible for the heat production hence lipolysis of adipocytes, we performed in vitro experiments. First, we prepared bone marrow-derived macrophages (BMDM) from myeloid-specific *Hif-1α* KO or WT mice and co-cultured them with 3T3-L1 adipocytes. We found that BMDM from myeloid-specific *Hif-1α* KO mice but not from WT mice were able to increase pHSL and UCP1 expression (Fig. 4D) and free fatty acid (FFA) release (Fig. 4E) from the co-cultured adipocytes. On the other hand, BMDM from *Ucp1* KO mice, which did not exhibit any changes in the ERthermAC fluorescence intensity (Supplementary Fig. 2D) failed to increase FFA release from the adipocytes (Fig. 4E). We then generated UCP1 overexpressing (OE) RAW264.7 macrophages (Fig. 4F) and were able to observe a significantly dimmer fluorescence intensity of ERthermAC in these macrophages (Fig. 4G). By co-culturing these UCP1 OE macrophages with 3T3-L1 adipocyte, we observed increased pHSL and UCP1 expression (Fig. 4H) and FFA release (Fig. 4I) from the adipocytes, results consistent with BMDM from myeloid-specific *Hif-1α* KO mice. We then tested whether the heat itself was sufficient to mediate lipolysis. To do this, we incubated 3T3-L1 adipocytes at 41 °C [43] for 4 h and found that this condition resulted in a significantly increased pHSL expression (Fig. 4J) and FFA release (Fig. 4K).

### Increased acetyl-CoA in HIF-1α-deficient ATM can boost the heat production

**Fig. 5:**
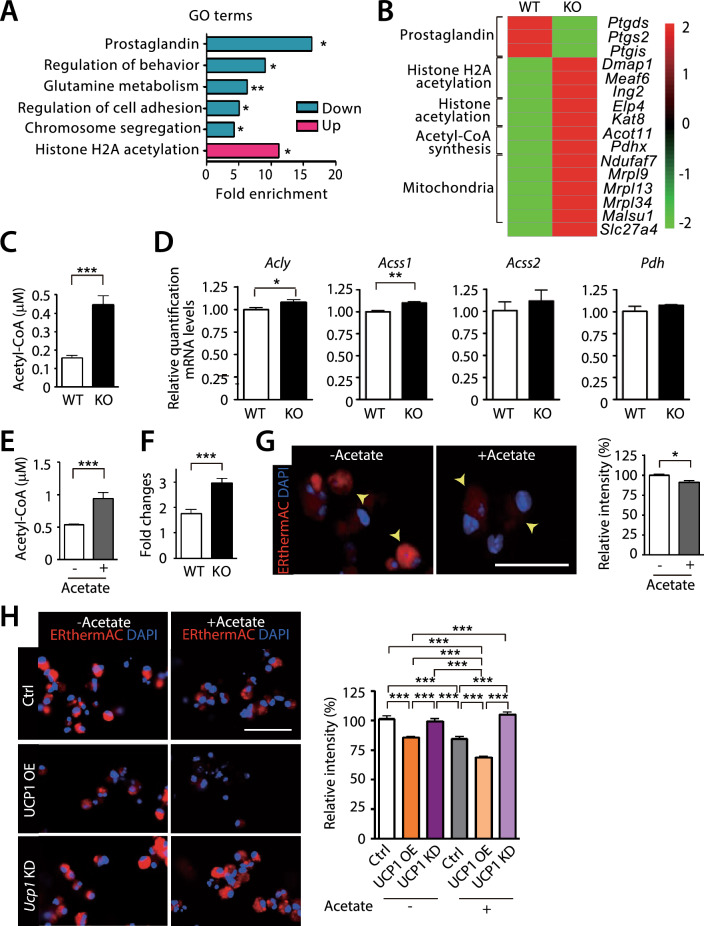
Increased acetyl-CoA in ATM deficient for HIF-1α may be responsible for the increased heat production. **A** Gene ontology (GO) analysis in the biological process of RNA sequencing results using FACS sorted ATM from WT or myeloid-specific *Hif-1α* KO mice fed with HFD for 12 wk. **B** Heat map of individual gene expression from RNA sequencing results in (A). **C** Acetyl-CoA production from the sorted ATM of WT or myeloid-specific *Hif-1α* KO mice fed with HFD. **D** mRNA levels of genes in FACS sorted ATM from WT or myeloid-specific *Hif-1α* KO mice fed with HFD. **E** Acetyl-CoA production from WT BMDM treated with vehicle or sodium acetate (5 mM). **F** Fold changes in acetyl-CoA levels in BMDM of WT or myeloid-specific *Hif-1α* KO mice treated with sodium acetate compared to those with the vehicle control. **G** ERthermAC (red) staining of WT BMDM treated with vehicle or sodium acetate. Nuclei are shown in blue with DAPI counterstaining. Scale bar denotes 50 μm. Quantification of ERthermAC intensity is shown on the right. *, ** and *** indicate *P* < 0.05, 0.01 and 0.001, respectively, by Student’s *t*-test. **H** ERthermAC (red) staining of BMDM transfected with empty, UCP1 OE, or UCP1 siRNA (KD) vector treated with or without sodium acetate. Nuclei are shown in blue with DAPI counterstaining. Scale bar denotes 100 μm. Quantification of ERthermAC intensity is shown on the right. *** indicate* P* < 0.001 by one-way ANOVA. Symbols and error bars are determined with the mean ± S.E.M

To understand how HIF-1α-deficient ATM generated the heat, we performed RNA sequencing analysis using the sorted ATM from myeloid-specific *Hif-1α* KO or WT mice fed with HFD. We found that gene ontology (GO) terms of histone H2A acetylation pathway (Fig. 5A; Supplementary Table 2) along with individual genes involved in histone acetylation, histone H2A acetylation, acetyl-CoA synthesis, and mitochondria biogenesis (Fig. 5B) were significantly upregulated in ATM isolated from myeloid-specific *Hif-1α* KO mice. Because histone acetylation requires acetyl-CoA as a critical intermediate metabolite [44], we hypothesized that acetyl-CoA synthesis might be increased in ATM defective for HIF-1α. By measuring acetyl-CoA levels in the sorted ATM, we indeed observed that acetyl-CoA levels were significantly higher in ATM of myeloid-specific *Hif-1α* KO mice than those of WT mice (Fig. 5C). Consistent with this finding, we observed increased expression of genes involved in acetyl-CoA synthesis including *ATP citrate lyase* (*Acly*), an enzyme converting citrate to acetyl-CoA [45] and *Acyl-CoA synthetase short chain family member 1* (*Acss1*), an enzyme that converts acetate to acetyl-CoA in the mitochondria [45] in ATM of myeloid-specific *Hif-1α* KO mice (Fig. 5D). Expression of *Acss2*, an enzyme that converts acetate to acetyl-CoA in the cytosol and nucleus [46] or *pyruvate dehydrogenase* (*Pdh*), a critical enzyme importing pyruvate into the mitochondria [47] was comparable in between ATM isolated from myeloid-specific *Hif-1α* KO and WT mice (Fig. 5D). We then wished to investigate whether acetyl-CoA synthesis could promote the heat production in macrophages. To do this, we treated BMDM with acetate, a substrate for acetyl-CoA [48] and observed that acetyl-CoA production was increased in WT BMDM (Fig. 5E) and more so in BMDM from myeloid-specific *Hif-1α* KO mice (Fig. 5F) by acetate treatment. Next, we measured the heat production in acetate-treated BMDM by using ERthermAC and observed that these cells exhibited a significantly dimmer fluorescence (Fig. 5G), indicating that acetate can boost the heat production in macrophages. Treatment of acetate to UCP1 OE BMDM further dampened ERthermAC fluorescence signal (Fig. 5H) while UCP1 knocked down (KD) BMDM did not exhibit any changes in the fluorescence intensity (Fig. 5H), indicating that UCP1 was absolutely necessary for the heat production and that acetate could significantly boost the heat generation even in the presence of UCP1.

### Increased numbers of UCP1-expressing macrophages are also observed in WAT of the lean human individuals

**Fig. 6:**
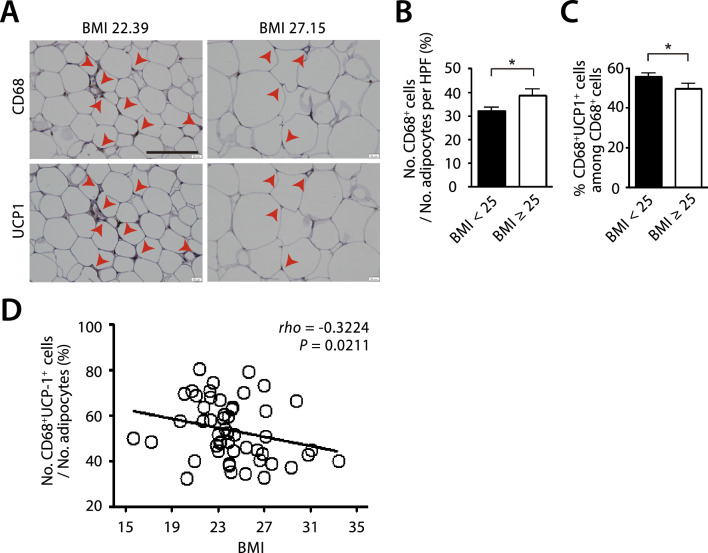
Increased UCP1-expressing macrophages in the lean individuals. **A** Immunohistochemistry staining of CD68 (upper) and UCP1 (lower) of omental WAT in human subjects. Red arrowheads indicate CD68- (upper) or UCP1-positive (lower) staining. Scale bar denotes 100 μm. Quantification of CD68^+^ cells normalized to the number of adipocytes per HPF (**B**) and CD68^+^UCP1^+^ cells among the total CD68^+^ cells (**C**) in patients with the body mass index (BMI) < 25 (n = 34) and BMI ≥ 25 (n = 17). Symbols and error bars in **B** and **C** are determined with the mean ± S.E.M. * and *** indicate *P* < 0.05 and 0.001, respectively, by Student’s *t*-test. **D** Correlation analysis between the percentage of CD68^+^UCP1^+^ cells and BMI of the human subjects

We next examined whether UCP1-expressing ATM existed in WAT of lean individuals. To do this, we obtained the omental adipose tissues from early-stage gastric cancer patients undergoing laparotomy surgeries and classified them to lean or obese based on their body mass index (BMI) with 25 being the cut off value (Supplementary Table 3). We observed the obese individuals (BMI ≥ 25) had increased numbers of CD68 + macrophages in WAT (Fig. 6A, B), consistent with the existing literature [49]. Interestingly and importantly, we observed the presence of UCP-1 expressing ATM in the lean individuals (BMI < 25) and found that the proportions of these cells were significantly increased than those in the obese individuals (Fig. 6A, C). Furthermore, there was a strong inverse relationship between the number of UCP1-expressing CD68 + ATM infiltrating WAT and BMI of the subjects (Fig. 6D).

## Discussion

Here, we demonstrate our novel findings that HIF-1α-deficient ATM could contribute to anti-obese phenotype by facilitating lipolysis of adipocytes through UCP1-mediated heat production. UCP1 has been known to be expressed in mostly BAT or WAT under the certain circumstances such as stimulation of the sympathetic nervous system releasing catecholamine acting on the β3-adrenergic receptor [30, 50]. In this study, we found that UCP1 can be expressed in ATM of WAT (Fig. 4A, B) and this did not require any stimuli but rather a targeted disruption of HIF-1 signaling.

How could HIF-1 regulate UCP1 expression in macrophages? Although we did not address this mechanism in the present study, it is possible that HIF-1 may activate molecules such as differentiated embryo-chondrocyte expressed gene 1 (DEC1), which in turn suppresses *PGC-1α* and *UCP1* at the transcription level as suggested by others [11, 51]. Consistent with these studies, we observed an increased expression of *Pgc1α* and *Ucp1* in ATM of myeloid-specific *Hif-1α* KO mice (Fig. 3D, 4A). Alternatively, since HIF-1 regulates some key enzymes involved in glycolysis [41], it may be likely that deletion of HIF-1α may increase PGC1α hence facilitating mitochondrial biogenesis [52] to compensate for the impaired glycolysis. In line with this, HIF-1α has been reported to regulate polarization of macrophages to M1 phenotype through induction of an aerobic glycolytic program that results in increased lactate production and intermediates of the Kreb cycle, producing various pro-inflammatory cytokines, reactive species, and nitric oxide [53]. Hence deletion of HIF-1α in macrophages would shift to M2 polarized metabolic profile with increased mitochondrial biogenesis through electron transport chain and fatty acid β-oxidation [53]. Indeed, we observed that *Hif-1α*-deficient ATM exhibited increased M2 phenotype with increased mitochondrial functions (Fig. 3).

Where does the energy source come from for UCP1-expressing ATM for the heat production? Although further studies are clearly warranted, we speculate that free fatty acid liberated from adipocytes may be taken up to ATM where they would then be converted to acetyl-CoA. In addition to glucose, fatty acid through β-oxidation can become an additional source of acetyl-CoA to facilitate tricarboxylic acid (TCA) cycle thereby enhancing UCP1-mediated heat production [54]. Interestingly, Zhao and Yue [55] have suggested that UCP1 expression on the inner mitochondrial membrane can soak up glucose and fatty acid uptake into cells (adipocytes in their study) in order to generate mitochondrial acetyl-CoA. In line with this, we also observed an increased acetyl-CoA level in ATM of the myeloid-specific *Hif-1α* KO mice (Fig. 5) and that an artificial increase in acetyl-CoA (induced by acetate treatment) was able to further increase the heat production from macrophages (Fig. 5). In mammalian cells, acetyl-CoA is produced within the mitochondria by ACSS1 and PDH, and in the cytosol and nucleus by ACLY and ACSS2 although these enzymes differ in their substrates in catalyzing reactions to synthesize acetyl-CoA [56]. Our observations that *Acly* and *Acss1* expression was increased in ATM of myeloid-specific *Hif-1α* KO mice compared to those of WT mice (Fig. 5D) may indicate that acetyl-CoA synthesis might be increased both in the mitochondria and nucleus to facilitate mitochondrial biogenesis and histone acetylation, respectively.

In this study we have utilized *ApoE* knockout mice to initially study the role of HIF-1α in myeloid cells in atherosclerosis. Despite the fact that this is a wildly used mouse strain for studying atherogenesis, it has been also reported that these mice are associated with increased proliferation of hematopoietic stem and multipotent progenitor cells, monocytes, and neutrophils [57]. This effect may have positively enforced our phenotype because MRP8 expression (thereby driving hMRP8 expression) has been reported to be on bone marrow myeloid progenitors to blood monocytes and neutrophils [58]. Although we have observed a similar result in the body weight gain of HFD-fed myeloid-specific *Hif-1α* KO mice in *ApoE* proficient background (Supplementary Fig. 1D) to those of *ApoE* knockout background (Fig. 1A), it clearly warrants further confirmation how ApoE might impact on HIF-1 signaling and UCP1 expression in ATM.

## Conclusions

We demonstrate that HIF-1α-deficient ATM produced a significant amount of heat through UCP1 expression, mediating lipolysis of white adipose tissues in mice. We believe that these findings provide a novel strategy to treat and prevent diet-induced obesity.

## Supplementary Information

Additional file 1.

Additional file 2.

## Data Availability

The data and information generated in this study are available within the article, Supplemental information, or upon reasonable request from the corresponding author.

## Abbreviations

ATM: Adipose-tissue macrophages
Adrβ: β-Adrenergic receptor
C/EBP: CCAAT/enhancer-binding protein
CREB: CAMP response-binding protein
eWAT: Epididymal white adipose tissue
FFA: Free fatty acid
HFD: High fat diet
HSL: Hormone-sensitive lipase
HIF: Hypoxia-inducible factor
iWAT: Inguinal white adipose tissue
KO: Knockout
NCD: Normal chow diet
PGC1α: Peroxisome proliferator activated receptor-gamma co-activator-1α
PPAR: Peroxisome proliferator-activated receptor
TG: Triglyceride
UCP1: Uncoupling protein 1
WT: Wild type
