# Supplementary figures and images for: Hypoxia-inducible factor-1α-deficient adipose-tissue macrophages produce the heat to mediate lipolysis of white adipose tissue through uncoupling protein-1
Source: Lab Anim Res. 2024 Oct 30;40:37. doi: 10.1186/s42826-024-00224-4 (PMC11523771; doi:10.1186/s42826-024-00224-4)

# Supplementary figure 1

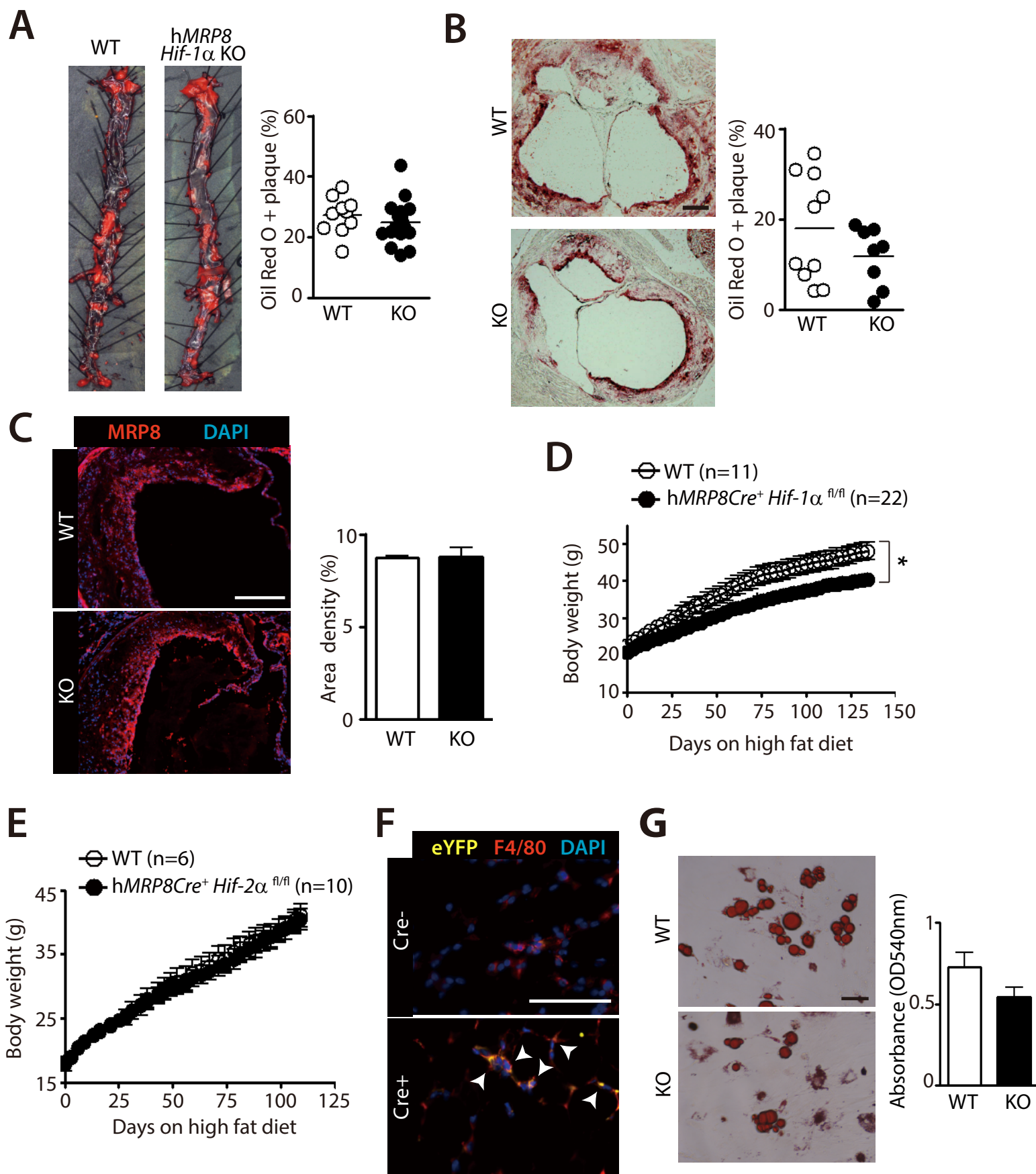

## Supplementary figure 2

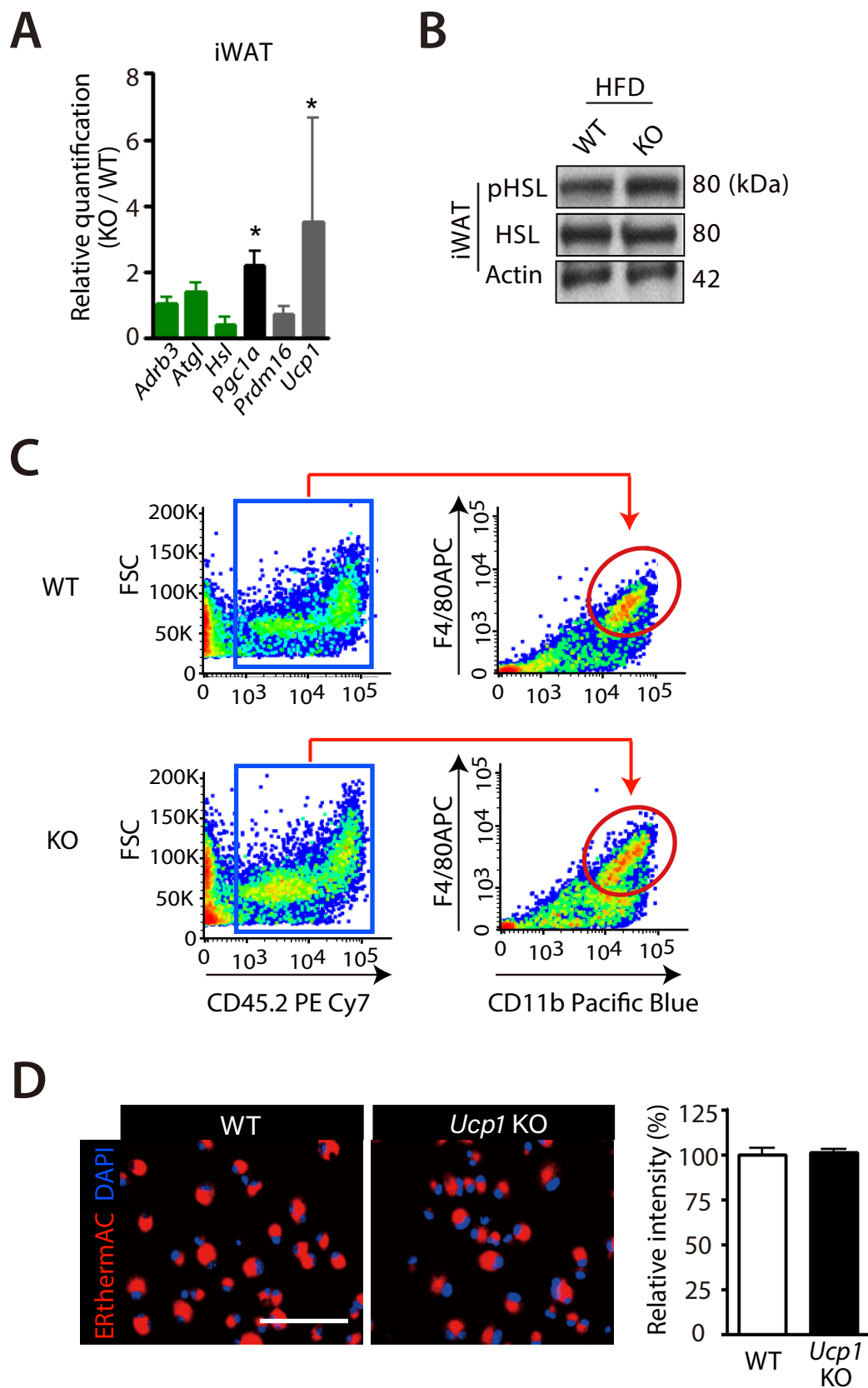

Supplement: Supplementary file 1 — Additional file 1. [file 42826_2024_224_MOESM1_ESM.pdf]
