# Supplementary material for: Hypoxia-inducible factor-1α-deficient adipose-tissue macrophages produce the heat to mediate lipolysis of white adipose tissue through uncoupling protein-1
Source: Lab Anim Res. 2024 Oct 30;40:37. doi: 10.1186/s42826-024-00224-4 (PMC11523771; doi:10.1186/s42826-024-00224-4)
Supplement: Supplementary file 2 — Additional file 2. [file 42826_2024_224_MOESM2_ESM.docx]

**Supplemental Information**

**Supplemental Figure Legends**

**Supplementary Figure 1.** (**A**) Representative images (left) and quantification (right) of *en face* analysis for Oil Red O staining of the aorta harvested from WT or *hMRP8cre*^+^;*Hif-1α*^fl/fl^;*ApoE*^-/-^ mice fed with HFD for 20 wk. Data are the mean ± s.e.m. (**B**) Representative images (left) and quantification (right) of Oil Red O staining of the aortic sinus harvested from mice in (A). (**C**) Immunostaining of the aortic sinus for myeloid cells using S100A8 (MRP8) antibodies (red). Nuclei are shown in blue with DAPI counterstaining. Scale bars in (B) and (C) denote 100 μm. Quantitative analysis of S100A8 positive area densities are shown in the bar graph (right) from at least 5 animals per group. (**D**) Body weight changes in WT and *hMRP8cre*^+^; *Hif-1α*^fl/fl^ mice fed with HFD. (**E**) WT and *hMRP8cre*^+^; *Hif-2α*^fl/fl^ mice fed with HFD. (**F**) Immunofluorescence staining of F4/80 (red) in eWAT isolated from ROSA-eYFP reporter mouse bearing hMRP8Cre-recombinase (Cre+; lower panel) or those not bearing Cre (Cre-; upper panel). Nuclei are shown in blue with DAPI counterstaining. White arrowheads indicate hMRP8 Cre-mediated YFP-positive cells that are co-localized with F4/80. Scale bar denotes 100μm. (**G**) Oil Red O staining of the pre-adipocyte of WAT of myeloid-specific *Hif-1α* KO or WT mice (n ≥ 5 mice per group). Scale bar indicates 100 μm. Symbols and error bars in (C), (D), and (E) are the mean ± S.E.M.

**Supplementary Figure 2.** (**A**) Relative difference of RNA expression in iWAT of *hMRP8cre*^+^; *Hif-1α*^fl/fl^; *ApoE*^-/-^. (**B)** Western blot of pHSL and HSL of inguinal WAT (iWAT) of WT or or *hMRP8cre*^+^; *Hif-1α*^fl/fl^; *ApoE*^-/-^ mice fed with HFD. Actin was used as a loading control. (**C**) Representative FACS plots of ATM analysis in WT or *hMRP8cre*^+^; *Hif-1α*^fl/fl^; *ApoE*^-/-^ mice fed with HFD for 12 wk. Red circles indicate total ATM. Daily feces (left) and urine (right) excretion from WT or *hMRP8cre*^+^; *Hif-1α*^fl/fl^; *ApoE*^-/-^ mice fed with HFD for 20 wk. (**D**) ERthermAC fluorescence images (red) and relative intensity (right) of BMDM obtained from WT or *Ucp1* KO mice. Scale bar indicates 50 μm. Symbols and error bars are determined with the mean ± S.E.M.

Supplemental Table 1. Primer sequences used for qRT-PCR analyses

| **Gene** | **Forward** | **Reverse** |
| --- | --- | --- |
| *β-actin* | CGAGCGTGGCTACAGCTTCA | AGGAAGAGGATGCGGCAGTG |
| *Cebpb* | GACAAGCTGAGCGACGAGTACA | CGACAGCTGCTCCACCTTCTTC |
| *Cebpd* | ACACGGGGTAAGGAGATGGA | CCTTCGGCAACCACCTAAAA |
| *Cebpa* | GCAAGAGCCGAGATAAAGC | TCAGGCAGCTGGCGGAAG |
| *Pparg* | AGACCACTCGCATTCCTTTG | TCGCACTTTGGTATTCTTGG |
| *Adrb1* | GGACTTCGGTAGATGTGCTGTGT | CGGTCCAGGGCGATGAC |
| *Adrb2* | ATGGGGCCACACGGGAACGA | GTGACGTGGTCTGGCGC |
| *Adrb3* | GGACGCTGTTCCTTTAAAAGCA | TCCATCTCACCCCCCATGT |
| *Atgl* | CACAGCGCTGGTCACTGGGG | CCGGGCCTCCTTGGACACCT |
| *Hsl* | AGACACCAGCCAACGGATAC | ATCACCCTCGAAGAAGAGCA |
| *Cpt1b* | CCCATGTGCTCCTACCAGAT | CCTTGAAGAAGCGACCTTTG |
| *Acox1* | GCCCAACTGTGACTTCCATC | GCCAGGACTATCGCATGATT |
| *Pgc1a* | CATTTGATGCACTGACAGATGGA | CCGTCAGGCATGGAGGAA |
| *Prdm16* | CAGCACGGTGAAGCCATTC | GCGTGCATCCGCTTGTG |
| *Ucp1* | CGATGTCCATGTACACCAAGGA | CCCGAGTCGGAGAAAAGAAG |
| *Pdk1* | GGACTTCGGGTCAGTGAATGC | TCCTGAGAAGATTGTCGGGGA |
| *Adfp* | CCT CAG CTC TCC TGT TAG GC | CAC TAC TGC TGC TGC CAT TT |
| *Srebf1c* | ACAAGATTGTGGAGCTCAAAGAC | GCGCAAGACAGCAGATTTATT |
| *Fas* | CCAGAGCCCAGACAGAGAAG | GACGCCAGTGTTCGTTCC |
| *aP2* | CATCAGCGTAAATGGGGATT | TCGACTTTCCATCCCACTTC |
| *Pdk4* | AGGCAAGACATCGGGTGG | CCTGGGTGAAGGGTTGACACT |
| *Resistin* | TTCCTTGTCCCTGAACTGCT | CCAATGTTCTTTATTGCATTTGG |
| *Leptin* | CAGGATCAATGACATTTCACACA | GCTGGTGAGGACCTGTTGAT |
| *Acaca* | GCCTCTTCCTGACAAACGAG | TGACTGCCGAAACATCTCTG |
| *Scd1* | TTCTTACACGACCACCACCA | GCAGGAGGGAACCAGTATGA |
| *Cd36* | GTCCTGGCTGTGTTTGGA | GCTCAAAGATGGCTCCATTG |
| *Lpl* | GGGCTCTGCCTGAGTTGTAG | CCATCCTCAGTCCCAGAAAA |
| *Ehhadh* | GGCTAGATGTGGGTTGGAAA | ACCAGCCCTTACCTGTCTTC |
| *Crat* | CTAACCTCCAACCACCGAAA | CCACCACCATGTAGCATCTG |
| *Glut1* | GCTGTGCTTATGGGCTTCTC | AGAGGCCACAAGTCTGCATT |
| *Cpt1a* | GGCATAAACGCAGAGCATTCCTG | CAGTGTCCATCCTCTGAGTAGC |
| *Adipoq* | AGATGGCACTCCTGGAGAGAAG | ACATAAGCGGCTTCTCCAGGCT |
| *Acly* | AAGGAGCCATTGTACCTGCC | ACTGGCAGGAATACTTGGGC |
| *Acss1* | TGCCTTCATTGTGCTGAAAG | TCCTCCAGGGTAGTGGTGTC |
| *Acss2* | TGGAGATGATCCTGTCACCA | GCATATGGCCACCTGTTTCT |
| *Pdh* | GTGAGAACAACCGCTATGGCATG | CGCAAACTTTGTTGCCTCTCGG |
| *Nd1* | CAGGATGAGCCTCAAACTCC | GGTCAGGCTGGCAGAAGTAA |
| *Sdh* | GGGCAAGCAACAGTATCTGC | GCACACAGGATGCACTCGTA |
| *Cytob* | ACGTCCTTCCATGAGGACAA | GAGGTGAACGATTGCTAGGG |
| *CoxII* | CCGTGGGGAATGTATGAGCA | CCAGGTCCTCGCTTATGATCTG |
| *Atp5b* | CACCACCAAGAAGGGATCGA | GCAGGGTCAGTCAGGTCATCA |
| *Tnfa* | CCAGACCCTCACTAGATCA | CACTTGGTGGTTTGCTACGAC |
| *Il6* | AACGATGATGCACTTGCAGA | GAGCATTGGAAATTGGGGTA |

**Supplemental Table 2**. **Gene Ontology (GO) of in the biological processes from the transcriptome analyses performed in FACS-sorted ATM.**

RNA sequencing data are available at GEO under accession number GSE143298. Gene lists in the biological process of up-regulated (≥ 3 fold) or down-regulated (≥ 3 fold) genes in ATM of WT or hMRP8 *Hif-1α* KO mice fed with 12 wks of high-fat diet. The data were analysed by DAVID version 6.7 (2016/05/24)

| GOTERM - Up | (*hMRP8*cre^+^;*Hif-1α*^fl/fl^;*ApoE*^-/-^ mice / WT mice) | | | |
| --- | --- | --- | --- | --- |
| Category | Term | Fold Enrichment | P-Value | Genes |
| GOTERM_BP_ALL | GO:0043968~histone H2A acetylation | 11.14341693 | 0.028381124 | RIKEN cDNA 2310005N01 gene; DNA methyltransferase 1-associated protein 1; inhibitor of growth family, member 3 |
|  |  |  |  |  |
| GOTERM - Down | (hMRP8cre^+^;*Hif-1α*^fl/fl^;ApoE^-/-^ mice / WT mice) | | | |
| Category | Term | Fold Enrichment | P-Value | Genes |
| GOTERM_BP_ALL | GO:0001516~prostaglandin biosynthetic process | 16.28129771 | 0.013705259 | prostaglandin D2 synthase (brain); prostaglandin I2 (prostacyclin) synthase; prostaglandin-endoperoxide synthase 2 |
| GOTERM_BP_ALL | GO:0046457~prostanoid biosynthetic process | 16.28129771 | 0.013705259 | prostaglandin D2 synthase (brain); prostaglandin I2 (prostacyclin) synthase; prostaglandin-endoperoxide synthase 2 |
| GOTERM_BP_ALL | GO:0006693~prostaglandin metabolic process | 11.62949836 | 0.026411526 | prostaglandin D2 synthase (brain); prostaglandin I2 (prostacyclin) synthase; prostaglandin-endoperoxide synthase 2 |
| GOTERM_BP_ALL | GO:0006692~prostanoid metabolic process | 11.62949836 | 0.026411526 | prostaglandin D2 synthase (brain); prostaglandin I2 (prostacyclin) synthase; prostaglandin-endoperoxide synthase 2 |
| GOTERM_BP_ALL | GO:0050795~regulation of behavior | 9.045165394 | 0.04233031 | artemin, prostaglandin D2 synthase (brain), thrombospondin 1;similar to thrombospondin 1 |
| GOTERM_BP_ALL | GO:0006541~glutamine metabolic process | 8.569104058 | 0.046749682 | asparagine synthetase domain containing 1; hypothetical protein LOC674493; cytidine 5'-triphosphate synthase; glutamine fructose-6-phosphate transaminase 2 |
| GOTERM_BP_ALL | GO:0009064~glutamine family amino acid metabolic process | 6.310580508 | 0.007808379 | aldehyde dehydrogenase 18 family, member A1; asparagine synthetase domain containing 1; hypothetical protein LOC674493; fumarylacetoacetate hydrolase; cytidine 5'-triphosphate synthase ;glutamine fructose-6-phosphate transaminase 2 |
| GOTERM_BP_ALL | GO:0045785~positive regulation of cell adhesion | 5.048464406 | 0.044044219 | secreted phosphoprotein 1; thrombospondin 1; similar to thrombospondin 1; integrin alpha 6 ;fibulin 2 |
| GOTERM_BP_ALL | GO:0007059~chromosome segregation | 4.239921279 | 0.029877157 | MIS12 homolog (yeast); SPC25, NDC80 kinetochore complex component, homolog (S. cerevisiae); stromal antigen 3; similar to nucleoporin 37; nucleoporin 37; RAD21 homolog (S. pombe) |

Supplemental Table 3. Information of the human subjects analyzed in Figure 7.

| **Patient number** | **Age** | **Gender** | **BMI** | **Cancer stage*** |
| --- | --- | --- | --- | --- |
| 1 | 42 | F | 20.11 | IA (pT1aN0) |
| 2 | 35 | F | 20.75 | IA (pT1aN0) |
| 3 | 58 | F | 21.14 | IA (pT1aN0) |
| 4 | 65 | F | 21.43 | IA (pT1aN0) |
| 5 | 76 | M | 22.29 | IA (pT1aN0) |
| 6 | 52 | M | 22.34 | IA (pT1bN0) |
| 7 | 53 | M | 22.39 | IA (pT1bN0) |
| 8 | 93 | M | 22.59 | IB (pT2N0) |
| 9 | 55 | M | 23.81 | IA (pT1bN0) |
| 10 | 53 | M | 26.65 | IA (pT1aN0) |
| 11 | 67 | M | 27.15 | IB (pT1bN1) |
| 12 | 61 | M | 27.15 | IA (pT1bN0) |
| 13 | 45 | M | 29.76 | IA (pT1aN0) |
| 14 | 63 | M | 30.78 | IIB (pT1bN3a) |
| 15 | 82 | M | 20.98 | IIIA (pT4aN2) |
| 16 | 57 | M | 25.45 | IIIB (pT4aN3a) |
| 17 | 62 | F | 23.25 | IB (pT1bN1) |
| 18 | 61 | F | 23.88 | IIIA (pT4aN1) |
| 19 | 59 | M | 24.37 | IA (pT1aN0) |
| 20 | 63 | M | 25.37 | IA (pT1bN0) |
| 21 | 64 | F | 23.98 | IIA (pT3N0) |
| 22 | 48 | F | 19.73 | IA (pT1aN0) |
| 23 | 80 | M | 23.53 | IIIB (pT4aN3a) |
| 24 | 59 | M | 33.44 | Tubular adenoma |
| 25 | 60 | F | 24.29 | IA (pT1bN0) |
| 26 | 61 | M | 23.19 | IA (pT1bN0) |
| 27 | 53 | F | 23.16 | IA (pT1bN0) |
| 28 | 46 | F | 21.76 | IA (pT1aN0) |
| 29 | 66 | M | 24.43 | IIIC (pT3N3b) |
| 30 | 78 | M | 24.23 | IIA (pT2N1) |
| 31 | 60 | F | 25.67 | IA (pT1aN0) |
| 32 | 73 | M | 26.98 | IB (pT2N0) |
| 33 | 74 | F | 23.88 | IA (pT1bN0) |
| 34 | 66 | M | 21.69 | IA (pT1bN0) |
| 35 | 61 | M | 23.04 | IB (pT2N0) |
| 36 | 35 | F | 20.34 | IA (pT1aN0) |
| 37 | 49 | M | 26.42 | IB (pT2N0) |
| 38 | 32 | M | 24.10 | IIIB (pT3N3a) |
| 39 | 78 | M | 25.22 | IA (pT1aN0) |
| 40 | 79 | M | 15.68 | IIA (pT2N1) |
| 41 | 46 | F | 17.2 | IIIB (pT3N3a) |
| 42 | 50 | F | 26.95 | IA (pTxN0) |
| 43 | 57 | M | 29.30 | IA (pT1aN0) |
| 44 | 68 | M | 31.06 | IA (pT1bN0) |
| 45 | 53 | M | 23.99 | IA (pT1aN0) |
| 46 | 71 | M | 23.58 | IIA (pT2N1) |
| 47 | 61 | M | 23.43 | IA (pT1bN0) |
| 48 | 58 | F | 26.85 | IA (pT1aN0) |
| 49 | 50 | F | 23.01 | IA (pT1bN0) |
| 50 | 68 | F | 27.62 | IA (pT1aN0) |
| 51 | 51 | F | 22.85 | IB (pT2N0) |

**^*^**Cancer stage was conferred according to AJCC (American Joint Committee on Cancer) 8^th^ edition

Supplemental Table 4. Average data on the human subjects in analyzed in Figure 7.

| Category  (number of patients) | Age (Mean ± s.e.m) | BMI (Mean ± s.e.m) |
| --- | --- | --- |
| Total (n = 51) | 59.9 ± 12.6 | 24.2 ± 3.3 |
| BMI < 25 (n = 34) | 59.7 ± 14.2 | 22.4 ± 2.0 |
| BMI ≥ 25 (n = 17) | 60.5 ± 8.7 | 27.8± 2.3 |
